# Proteomics alterations in chicken jejunum caused by 24 h fasting

**DOI:** 10.7717/peerj.6588

**Published:** 2019-03-26

**Authors:** Ádám Simon, Gabriella Gulyás, Zoltán Mészár, Mangesh Bhide, János Oláh, Péter Bai, Éva Csősz, András Jávor, István Komlósi, Judit Remenyik, Levente Czeglédi

**Affiliations:** 1 Department of Animal Science, Faculty of Agricultural and Food Sciences and Environmental Management, University of Debrecen, Debrecen, Hungary; 2 Department of Anatomy, Histology and Embryology, Faculty of Medicine, University of Debrecen, Debrecen, Hungary; 3 Laboratory of Biomedical Microbiology and Immunology, University of Veterinary Medicine and Pharmacy, Košice, Slovakia; 4 Slovak Academy of Sciences, Institute of Neuroimmunology, Bratislava, Slovakia; 5 Farm and Regional Research Institute of Debrecen, University of Debrecen, Debrecen, Hungary; 6 Department of Medical Chemistry, Faculty of Medicine, University of Debrecen, Debrecen, Hungary; 7 MTA-DE Lendület Laboratory of Cellular Metabolism, University of Debrecen, Debrecen, Hungary; 8 Research Center for Molecular Medicine, Faculty of Medicine, University of Debrecen, Debrecen, Hungary; 9 Proteomics Core Facility, Department of Biochemistry and Molecular Biology, Faculty of Medicine, University of Debrecen, Debrecen, Hungary; 10 Laboratory of Animal Genetics, Faculty of Agricultural and Food Sciences and Environmental Management, University of Debrecen, Debrecen, Hungary; 11 Institute of Food Technology, Faculty of Agricultural and Food Sciences and Environmental Management, University of Debrecen, Debrecen, Hungary

**Keywords:** 2D-DIGE, Chicken, Expression, Fasting, qPCR, Small intestine, Morphometry, Villus, mRNA, Protein

## Abstract

The small intestine is the longest part of the chicken (*Gallus gallus*) gastrointestinal system that is specialized for nutrient absorption. It is known that decrease in intestinal villi area or height in early age can cause a reduction in essential nutrient intake, which may lead to delayed growth and consequently poorer performance of broiler chickens. The small intestinal absorptive surface is known to be affected by various factors, among others things the nutritional state. In our experiment, we aimed to investigate the possible protein expression alterations that lie behind the villus area and height decrease caused by feed deprivation. A total of 24 chickens were divided into three groups, namely ad libitum fed, fasted for 24 h, fasted for 24 h then refed for 2 h. The morphometric parameters were also measured in the duodenum, jejunum and ileum tissue sections using image analysis. Differential proteome analyses from jejunum samples were performed using two-dimensional difference gel electrophoresis followed by tryptic digestion and protein identification by matrix-assisted laser desorption/ionization mass spectrometry. Overall 541 protein spots were detected after 2D. Among them, eleven showed 1.5-fold or higher significant difference in expression and were successfully identified. In response to 24 h fasting, the expression of nine proteins was higher and that of two proteins was lower compared to the ad libitum fed group. The functions of the differentially expressed proteins indicate that the 24 h fasting mainly affects the expression of structural proteins, and proteins involved in lipid transport, general stress response, and intestinal defense.

## Introduction

The small intestine is the longest part of the chicken gastrointestinal system (GIT). It is specialized for nutrient absorption and can be divided into duodenum, jejunum and ileum. Glucose and vitamins are mainly absorbed in the duodenum, amino acids in the jejunum and fatty acids in the distal part of the jejunum and in the start of the ileum (Denbow, 2012). For efficient nutrient absorption, intestinal villi are required to increase the surface area of the intestinal wall. The villus is a finger like projection of the intestinal mucosa, a constantly regenerating structure with a life cycle of 4 days. The intestinal mucosa’s epithelium contains the brush border (consisting of microvilli), which further increases this absorptive surface.

The small intestinal absorptive surface is known to be either positively or negatively affected by various factors: including the environment (Santos et al., 2015; Zhang et al., 2015), feed supplements from various sources (Xu et al., 2003; Laudadio et al., 2012; Yang et al., 2017) pathogenic microbiota and mycotoxin feed contaminants (Jayaraman et al., 2013; Yu et al., 2018). It is already known to be altered by different nutritional conditions (Yamauchi, Kamisoyama & Isshiki, 1996; Bhanja et al., 2009). Fasting can cause a decrease in the villi area, crypt number and depth, number of proliferating cells and reduction in the length of the entire small intestinal segment. Reports have shown that fasting hatched chicks for 48 h (when the yolk sack becomes empty) or longer causes a decrease in performance later in life (Gonzales et al., 2003; Careghi et al., 2005). Decreased intestinal absorption area limits the uptake capacity of essential nutrients for optimal growth, thus contributes to decreased body weight at slaughter age and increases mortality during the growth period (Geyra, Uni & Sklan, 2001). These results suggest a maximum fasting of 24 h after hatching in order to preserve broiler performance at market age (42 days). Day long fasting is also important in later life stages in case of broiler chickens to improve feed efficiency and to reduce excessive abdominal fat (Oyedeji & Atteh, 2005; Leeson & Summers, 2009) and also in case of layer chickens to force molting (Berry, 2003). It is also became evident that fasting can cause a reduction in the intestinal absorptive surface (i.e., villus area) in later life too (45 days) which can be reversed by 24 h refeeding (Shamoto & Yamauchi, 2000).

Currently there is a lack of information on the alterations in the protein expression that lies behind the already observed small intestinal morphometric changes caused by fasting. We aimed to identify these using two-dimensional difference gel electrophoresis (2D-DIGE) under ad libitum fed and fasted conditions in the jejunum as it is the main site of nutrient absorption in chickens (Denbow, 2012).

## Materials and Methods

### Ethical approval

The experiment followed the European Union principles for animal care and experimentation (EC Directive 86/609/EEC) and it was approved by the local Committee of Animal Welfare, University of Debrecen (registration number: DEMAB/12-7/2015).

### Birds and experimental conditions

Experimental setting was the same as described in Simon et al. (2018). Briefly, 24 broiler type (Ross 308 line, all 28-day-old), chickens were allocated to three experimental groups (with eight bird/group), namely ad libitum fed (ad libitum group); fasted for 24 h (F24h group); fasted for 24 h then refed for 2 h (F24hRF2h group).

### Intestine morphometric analysis

All chickens were terminated by concussion as recommended in 40/2013. (II. 14.) Hungarian act about animal experiments. Intestinal samples were collected within 10 min after euthanasia. The *duodenum* (middle part), *jejunum* and *ileum* (both one cm from Meckel’s diverticulum) samples (*n* = 3 per group) were obtained from the three experimental group (ad libitum fed, F24h, F24hRF2h). Intestinal content was flushed out with 1× PBS (2.7 mM KCl, 1.8 mM KH_2_PO_4_, 10 mM Na_2_HPO_4_, 137 mM NaCl, pH: 7.4) and then fixed in 10% neutral-buffered formalin solution. Tissue sections were stained with hematoxylin and eosin according to Fischer et al. (2008) and digitalized with a Leitz Diaplan microscope (Spot RT-Slider; Diagnostic Instruments, Sterling Heights, USA). Morphometric measures were performed using Photoshop CC 2015 1.2 software (Adobe Systems Inc., San Jose, CA, USA) according to Applegate et al. (1999). First, we measured the scale bar with the ruler tool, then we set a custom measurement scale (510 px = one mm). Finally, the depth of crypts (depth of mucosal invaginations between adjacent villi), the width of *muscularis externa* and the area and height of villi were recorded (25 technical measurement on each tissue section image).

### Protein isolation and fluorescent labeling

Frozen jejunum samples (*n* = 8, from each group) were crushed to fine powder with mortar and pestle in liquid nitrogen. A total of 50 mg of this powdered sample was weighed with an analytical balance in tubes with low protein affinity (Low-bind; Eppendorf, Hamburg, Germany). Then 500 μl cold lysis buffer (7 M urea, 2 M thiourea, 4% CHAPS, 30 mM Tris, pH: 8.8) was added to each one. Samples were placed on ice for 1 h and vortexed at every 10 min (10 s for each). After lysis, homogenates were centrifuged at 16,000×*g* for 30 min at 4 °C (Centrifuge 5810 R; Eppendorf, Hamburg, Germany). Supernatants were collected to fresh tubes and the protein concentration was determined with RC DC Protein Assay kit (Bio-Rad, Hercules, CA, USA). After the protein precipitation and centrifugation steps the supernatant was decanted and the precipitates were washed twice to ensure detergent compatibility as recommended by the kit. Bovine serum albumin (Sigma-Aldrich, St. Louis, MO, USA) was used as a concentration standard. Absorbance data were collected with a SPECTROstar Nano (BMG Labtech, Ortenberg, Germany) microplate reader at 650 nm. Sample concentrations were interpolated from a standard curve using MARS data analysis software 3.10 (BMG Labtech, Ortenberg, Germany). The protein concentrations of the lysates ranged between 5.32 and 11.11 μg/μl (Table S1). Supernatants were stored at −70 °C for further use. Prior to the fluorescent labeling pH of the samples were checked with Universal Indicator paper (Rota; VWR, Poole, England) to ensure that all are in the range of pH: 8–9. For each gel 50 μg of sample pool (a mixture of all sample, for normalization) was labeled with 400 pmol of Cyanine2 NHS ester minimal dye (Lumiprobe, Hannover, Germany) and 50 μg from ad libitum and 50 μg from F24h group with each of 400 pmol of Cyanine3 or Cyanine5 using dye swap method (Table 1). Labeling reactions were carried out on ice for 30 min in dark and then 10 mM lysine stop solution was added to each reaction and incubated on ice for 10 min.

**Table 1:** Samples labeled with dye swap method.

	Fluorescent dye		
Gel	Cy2	Cy3	Cy5
1	Pool^†^	A^‡^-1	F^§^-8
2	Pool	F-7	A-2
3	Pool	A-3	F-6
4	Pool	F-5	A-4
5	Pool	A-5	F-4
6	Pool	F-3	A-6
7	Pool	A-7	F-2
8	Pool	F-1	A-8

**Notes:**

† A mixture containing each sample.

‡ Sample from ad libitum group.

§ Sample from fasted for 24 h group.

### Two-dimensional difference gel electrophoresis and image analysis

For the first dimension, seven cm immobilized linear pH gradient strips (pH: 5–8, Bio-Rad, Hercules, CA, USA) were used. We decided to use pH range five to eight as our previous tests showed the majority of proteins in this tissue can be detected in this region. Strips were rehydrated by passive rehydration (using 150 μg of labeled protein) dissolved in 125 μl rehydration buffer (2 M thiourea, 8 M urea, 2% CHAPS, 0.2% (v/v) Bio-Lyte 4/6 and 6/8 ampholyte at a ratio of 1:2, 0.002% (w/v) bromophenol blue) for 16 h at room temperature. Isoelectric focusing was conducted in Protean IEF Cell (Bio-Rad, Hercules, CA, USA). Low voltage (250 V) was applied for 20 min and then, the voltage was gradually increased to 4,000 V over 2.5 h and maintained at that level until a total of 15,349 Vh. Focused IPG strips were equilibrated for 10 min at 50 rpm on orbital shaker (Biosan, Riga, Latvia) in 6 M urea, 20% (v/v) glycerol, 2% (w/v) SDS, 50 mM Tris pH: 8.8 and 2% (w/v) DTT and then for an additional 10 min in the same buffer except that DTT was replaced by 2.5% (w/v) iodoacetamide. After equilibration, proteins were separated by molecular mass using OmniPAGE Mini (Cleaver Scientific, Rugby, UK) vertical electrophoresis system. Electrophoresis was performed on 100 × 100 × 1 mm, 13% polyacrylamide gels (37.5:1 acrylamide:bis-acrylamide ratio) in 1X TGS electrophoresis buffer containing 25 mM Tris, 192 mM glycine and 0.1% (w/v) SDS. The gels were run by applying 90 V until samples entered from the stacking agarose gel (0.5% agarose in 1× TGS) to the separation gel and then 180 V until the tracking dye reached the end of the gels. Preparative polyacrylamide gels for spot picking (containing 500 μg total protein) were stained with colloidal Coomassie G-250 (Thermo Fisher Scientific, Waltham, MA, USA) using a protocol described by Dyballa & Metzger (2009). Gel images were recorded using PharosFX Plus (Bio-Rad, Hercules, CA, USA) scanner with external laser module at 100 μm resolution (254 dpi) using Quantity One 29.0 (Bio-Rad, Hercules, CA, USA) data collector software and exported as 16-bit .tif files (Fig. S1). The images were analyzed with Delta2D 4.3 software (Decodon™ GmbH, Greifswald, Germany). Spots were detected, quantified and normalized according to the volume ratio of corresponding spots detected in the Cy2 image (internal standard) using the in-gel standard warping strategy. Warping is necessary to eliminate the distortions between the gels caused by the run to run differences in electrophoresis in order for proper spot detection. Using the in-gel standard warping strategy, first the sample images (Cy3–Cy5 channels) were distorted to the standard image (Cy2 channel), then all the standard images to a selected standard image.

### Protein identification

Spots of interest were cut out from the Coomassie-stained preparative gels and digested according to Shevchenko et al. (2006). Digested samples were prepared to mass spectrometry (MS) in the same way as described by Bhide et al. (2009). Matrix-assisted laser desorption/ionization (MALDI)-MS data were obtained in an automated analysis loop (Ultraflex; Bruker-Daltonics, Leipzig, Germany) equipped with a LIFT-MS/MS device. Spectra were acquired in the positive-ion mode at 50 Hz laser frequency and 100–1,000 individual spectra were averaged. Automated analysis of mass data was performed with FlexAnalysis software (Bruker-Daltonics). MALDI-MS and MALDI-MS/MS data were combined (BioTools; Bruker-Daltonics) to search a non-redundant protein database (*Gallus gallus*, NCBInr) using Mascot software (Matrix Science, Boston, USA) using the following search parameters: peptide mass fingerprint search, trypsin digestion, unrestricted monoisotopic mass values, 1+ peptide charge state, ± 1.2 peptide mass tolerance and with at maximum one missed cleavages sites.

### RNA isolation, reverse transcription, qPCR assays

Whole jejunum samples (eight birds per group) were ground in liquid nitrogen using a mortar and pestle. Powdered tissue (25 ± 2.5 mg) was lysed in TRI Reagent solution (Thermo Fisher Scientific, Waltham, MA, USA), with an Ultra-Turrax T10 at setting five for 1 min. Total RNA was isolated using Direct-zol™ RNA MiniPrep (Zymo Research, Orange, CA, USA) using an on-column DNase I digestion step (using 30 U enzyme for 15 min). Isolated RNA amounts and purity were quantified in eluent using a NanoDrop ND-1000 spectrophotometer (Thermo Fisher Scientific, Waltham, MA, USA). RNA integrity was checked with 1% agarose gel electrophoresis. Reverse transcription was conducted using 500 ng total RNA with qPCRBIO cDNA Synthesis Kit (PCR Biosystems, London, UK). Reaction conditions were as recommended by the kit’s manual. Prior to qPCR, cDNA samples were diluted 10-fold, then stored in −20 °C until qPCR. Assays were conducted as described by Simon et al. (2018). Reference gene with stable expression (*TBP*) was revealed from five candidates (Table S2) and used for normalization as a single reference gene as suggested by the reference gene testing method. Details of qPCR primers were specified in Table 2.

**Table 2:** Details of primers and amplicons used to measure the mRNA expression of the identified proteins.

Gene	Sequence accession number	Primers (5′–3′ sequence of forward and reverse)	Reaction efficiency (E)^†^	Amplicon length (bp)	Amplicon length on gDNA (bp)	Amplicon *T*_m_ (°C)^‡^
*MUC6*	XM_015286750.1	AAGCTTGGGCAGAAAAGACATGTT	1.883	105	1,287	88.2
		AGTCTCGGACACAGGCCTCA				
*HSP90AA1*	NM_001109785.1	CTGAAGGACTACTGCACTCGCAT	1.943	81	907	80.3
		AGCCACCTGGTCCTTTGTCTCAC				
*ACTA2*	XM_015288392.1	ATGGCCAGGTCATCACCATTGGA	1.933	101	1,068	83.7
		GGTTTCATGAATGCCAGCGGACT				
*KRT14*	NM_001001311.2	AGCAGGAGATCGCCACGTACC	1.918	94	439	81.0
		CTGCCAGAGTGTGAGGACATTGC				
*APOA5*	XM_417939.5	CTGCGCTTCTGCTCACCCT	1.974	79	562	83.3
		AGGTACTCCCAGAAGCCACTCC				
*MAGT1*	NM_001006435.1	AAGAACCCCCACACAGGACAAGT	1.934	63	2,451	83.0
		TGCAACAAACTGGGCTTGGCT				
*APOA1*	NM_205525.4	CTGGTGACCCTCGCTGTGCTC	1.896	107	328	86.6
		ACGTCCACCATATCCCGAATGC				
*TPM1*	NM_205401.1	GAGACCCGTGCTGAATTTGCT	1.935	98	7,133	81.6
		AGGTTTTCTTCTTTGGCATGCG				
*CHP1*	NM_001007930.1	AAGCTGATCAAGATGGGGACT	1.940	82	1,325	80.4
		ATTTTCTGCTCTACGTCCACCT				
*EXFABP*	NM_205422.1	CTAGGGAGCGGAACTACACGGAT	1.972	107	908	77.0
		TTGTTTGGGAAGCAGCATTCATC				
*ACTB*^§^	NM_205518.1	AGATCACAGCCCTGGCACCTAG	1.880	61	416	80.9
		TTGCGCTCAGGTGGGGCAAT				
*TBP*^§^	NM_205103.1	ATCAAGCCAAGAATTGTTCTGC	1.945	85	981	78.2
		CTTCGTAGATTTCTGCTCGAACT				

**Notes:**

† Determined with LinRegPCR.

‡ Determined with melt curve analysis.

§ Designed in Simon et al. (2018).

### Statistical analysis

Statistical analysis of protein spot intensities and qPCR data was performed with GraphPad Prism 7 (La Jolla, CA, USA) software using two-sided, unpaired *t*-test. Morphometric data was analyzed with one-way ANOVA, followed by Dunn’s *post hoc* test to compare every mean with each other. Data from qPCR analysis were expressed as group means normalized to mRNA levels of ad libitum fed samples. Results with *P* < 0.05 were considered as statistically significant.

## Results

### Small intestine morphology

No significant change was observed between the groups in all measured parameters. One exception was the ileal villus area of the birds that were fasted for 24 h then refed for 2 h. In that case a significant 34% increase was observed compared to the villus area of the birds in the ad libitum fed group (Table S3).

### Proteome analysis

**Figure 1:**
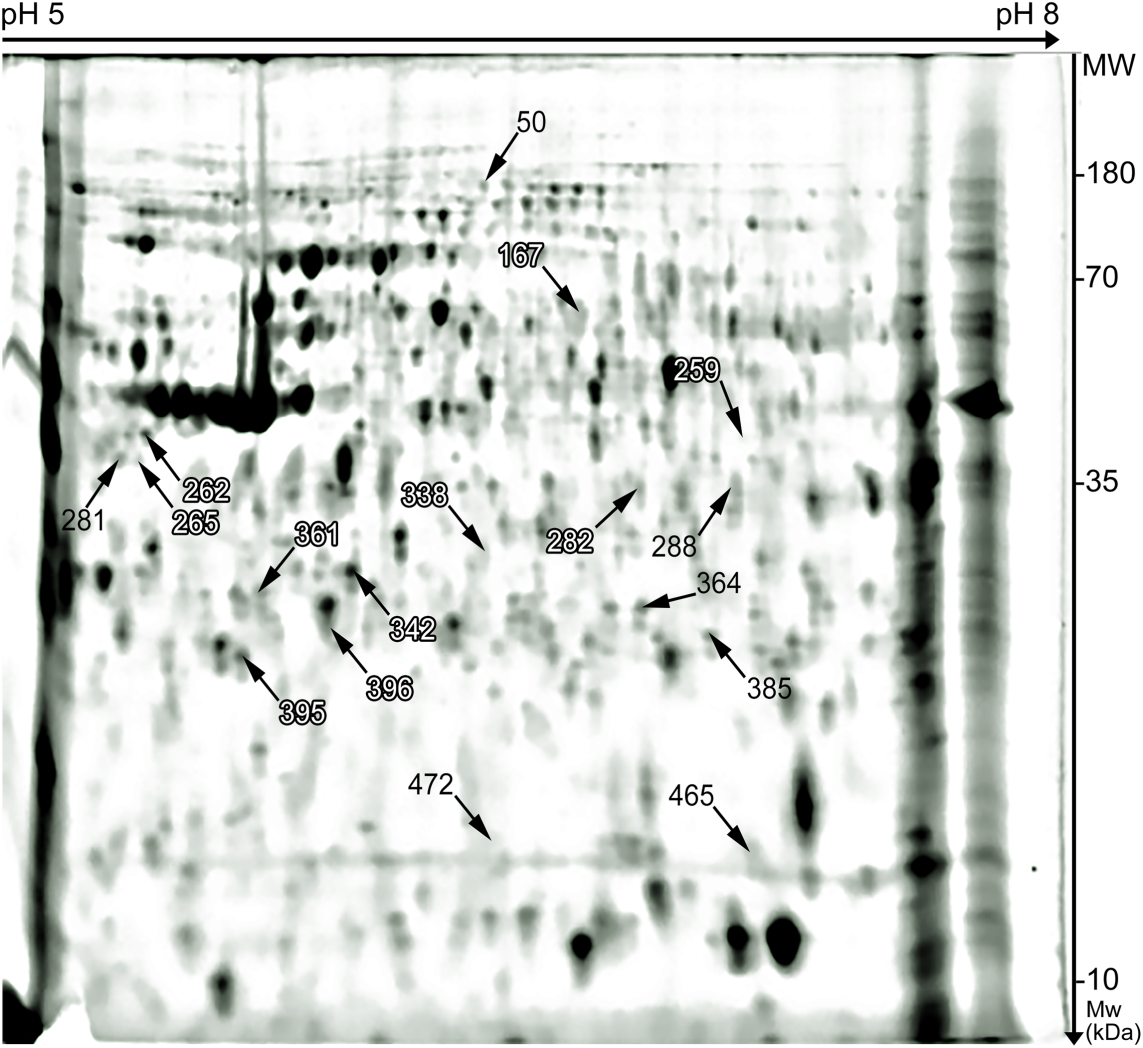
Fusion gel image of 2D DIGE experiment containing all protein spots (541) in chicken jejunum created with Delta2D software. Proteins with higher expression in the fasted for 24 h group compared to the ad libitum fed group are marked with black and proteins with lower expression are labeled with white numbers, respectively. Molecular weight marker (PageRuler 10–180 kDa, Thermo Fisher Scientific, Waltham, MA, USA) was derived from the preparative gel where it was run together with the sample proteins.

**Figure 2:**
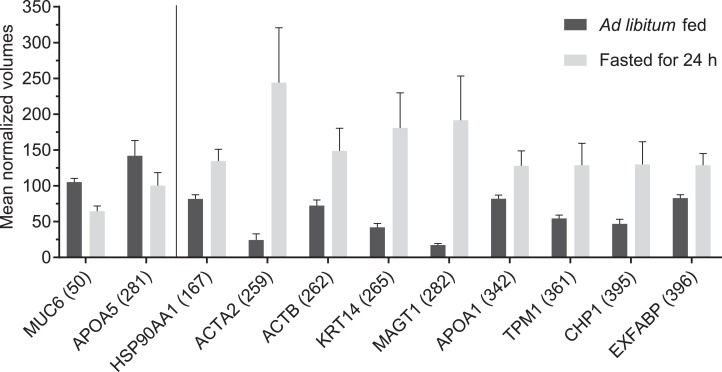
Mean normalized volumes of significantly (*P* < 0.05) differentially expressed, successfully identified spots. Data are presented as group means, error bars represents ± SEM (with *n* = 8 in each group). The vertical line separates between downregulated (left) and upregulated (right) protein spots compared to the ad libitum fed group.

After performing the 2D-DIGE experiments, approximately 541 spots were detected on each polyacrylamide gel (Fig. 1). Among them 88 showed statistically significant difference between two experimental groups (ad libitum fed group vs. fasted for 24 h group). After setting a 1.5-fold change cut-off value, seventeen spots showed significant difference in intensity between the two groups (Fig. 2) and sent to MS. Finally, 11 proteins were identified successfully (Table 3).

**Table 3:** Successfully identified, differentially expressed proteins in chicken jejunum between ad libitum and 24 h fasted groups.

Spot ID	Accession number (*Gallus gallus*)	Identified protein	Encoding gene	N/C^†^	Fold-change^‡^	*P*-value	MW (kDa)^§^	pI (pH)^§^	Score^#^
50	F1NBL0	Mucin-6	*MUC6*	17/12	−1.63	<0.001	132	5.78	142
167	P11501	Heat shock protein 90 kDa alpha class A member 1	*HSP90AA1*	11/14	1.65	0.009	84	5.01	116
259	P08023	Actin, smooth muscle	*ACTA2*	17/52	9.97	0.013	33	5.46	240
262	P60706	Actin, cytoplasmic 1	*ACTB*	18/41	2.05	0.034	41.7	5.29	232
265	Q6PVZ1	Keratin, type I cytoskeletal 14	*KRT14*	19/20	4.32	0.014	51.0	5.02	185
281	XP_417939	Apolipoprotein A5	*APOA5*	21/34	−1.54	0.044	40.3	6.26	239
282	Q5ZJ06	Magnesium transporter protein 1	*MAGT1*	10/19	11.07	0.013	36.7	9.66	122
342	P08250	Apolipoprotein A1	*APOA1*	16/31	1.57	0.048	30.7	5.58	202
361	P04268	Tropomyosin alpha-1 chain	*TPM1*	19/32	2.36	0.031	32.8	4.7	207
395	Q5ZM44	Calcineurin B homologous protein 1	*CHP1*	21/79	2.77	0.022	22.4	4.97	395
396	P21760	Extracellular fatty acid-binding protein	*EXFABP* (*CALII*)	9/34	1.56	0.017	20.2	5.56	116

**Notes:**

† Number of matched peptides/sequence coverage percentage (%).

‡ Protein expression of fasted group compared to the ad libitum group.

§ Theoretical molecular mass (MW) and isoelectric point (pI).

# Protein score is −10 * Log (*P*), where *P* is the probability that the observed match is a random event. Mascot scores greater than 63 were considered significant (*P* < 0.05).

### The identified proteins

#### Actin, cytoplasmic and smooth muscle type (ACTB, ACTA2)

The α-actin (smooth muscle type) is the building element of thin filaments. The β- and γ-actins (cytoplasm types) are present in most of eukaryote cells and takes part in the cytoskeleton assembly. The smooth muscle type actin can be found in the chicken small intestine smooth muscle layer, where it is responsible to maintain the small intestine motility (Dehkordi, Baghai & Rahimi, 2016). In the microvilli, dense bundles of actin filaments can be found, which are cross linked with villin and fimbrin proteins. These bundles are fixed to the cell membrane with myosin-1 (Fath & Burgess, 1995). In our experiment both smooth muscle and cytoplasmic type actin showed increased expression by 9.97- and 2.05-fold, respectively, in the fasted group.

#### Apolipoprotein A1 and A5

The apolipoproteins are lipid binding proteins, which are responsible for lipid transport through lymph and blood circulation. Avian apolipoproteins are expressed in numerous tissues including brain, liver, skeletal muscle, intestine (Byrnes et al., 1987; Dichlberger et al., 2007) The apolipoprotein A1 (APOA1) is the main protein constituent of the high-density lipoprotein (HDL), which transport cholesterol from tissues to the liver (reverse lipid transport). A total of 1.57-fold increase was observed in APOA1 protein level compared to ad libitum state. The apolipoprotein A5 (APOA5) is presents in very low concentrations in plasma compared to other apolipoproteins. This suggests a local catalytic role for APOA5 in reducing blood plasma triglyceride level by activating lipoprotein lipase instead of lipid transport through circulation (Hubacek, 2016; Nilsson et al., 2011). A total of −1.54-fold decrease in APOA5 protein was recorded after 24 h fasting in the chicken jejunum.

#### Calcineurin homologous protein 1

The calcineurin homologous protein 1 (CHP1) has calcium-ion binding properties and can be found in the polarized cell membranes (like the intestinal epithelium). The CHP1 is also colocalized with the exocytotic vesicles and proposed to be involved in controlling vesicle movement along microtubules by interacting with kinesin (Matsushita et al., 2007; Di Sole et al., 2012). Currently no results can be found in the scientific literature investigating a connection between increased expression of CHP1 and the effect of fasting. We found a 2.77-fold increase in CHP1 levels in fasted broilers, compared to ad libitum fed group.

#### Extracellular fatty acid-binding protein

This protein is secreted to the extracellular space and has fatty acid binding and siderophore properties, which suggest a defensive role against *Escherichia coli* and *Salmonella* species in chickens (Correnti et al., 2011; Rychlik, Elsheimer-Matulova & Kyrova, 2014). Without extracellular fatty acid-binding protein (EXFABP), chicken liver cells show dramatic reduction in viability and differentiation ability with increased apoptosis (Di Marco et al., 2003). Increased (1.56-fold) EXFABP expression may be required to stimulate cell proliferation, tissue repair and defense against bacterial infection in case of altered intestinal morphology after fasting.

#### Heat shock protein 90 kDa alpha class A member 1

The HSP90A is the stress inducible isoform of HSP90, while HSP90 beta has constant expression. The HSP90 can be found in every eukaryote cell and represents 4–6% of total protein content in stressed cells (Zuehlke et al., 2015). The protein level of HSP90 alpha increased by 1.65-fold in response to fasting.

#### Keratin, type I cytoskeletal 14

The chicken genome has threefold higher keratin encoding gene repertoire (scales, claws and feathers) compared to mammals. The intermediate filament keratin 14 (K14) belongs to the cytokeratin family. Together with two type II keratin (K5) molecules, it forms the intermediate filaments part of the cytoskeleton in epithelial cells (Hillier et al., 2004; Schweizer et al., 2006). The K14 was already identified in rabbit duodenum along with actin in the crypt and villus cells and seems that K5/K18, K7/K17 and the K8/K14 filament pairs are the most important molecules forming the villus structure (Iwatsuki & Suda, 2005, 2010) in these tissues. In case of domestic chicken, the expression of different keratin types in the jejunum are currently uncharacterized. In our experiment we measured a 4.32-fold increase in K14 expression in the fasted group.

#### Magnesium transporter protein 1

The magnesium transporter protein 1 (MAGT1) is one of the cell membrane’s Mg^2+^ channels, which take part in the maintaining of intracellular magnesium level. Currently there is a lack of information about the nature of MAGT1 in birds, but it is expressed in every mammalian tissue type (Zhou & Clapham, 2009). In the mouse kidney and sheep rumen epithelial cells MAGT1 expression is known to be altered in response to changing extracellular magnesium concentrations (Goytain & Quamme, 2005). Increase in its expression (11.07-fold) maybe in connection with the lack of Mg^2+^ in the chicken intestinal lumen caused by fasting.

#### Mucin-6

The mucins are large, highly glycosylated proteins. These proteins are forming the mucosal surface of the gastrointestinal tract. The mucosal layer protects against the digestive enzymes and pathogens and also helps to forward the intestinal content. The chicken genome encodes three transmembrane type mucins (MUC4, MUC13, MUC16) and four secreting, gel forming type (MUC2, MUC5AC, MUC5B, Mucin-6 (MUC6)) identified by Lang, Hansson & Samuelsson (2006). A −1.63-fold change was observed in MUC6 level compared to the control group.

#### Tropomyosin alpha-1 chain

Tropomyosin can be found in muscle and non-muscle type cells. In non-muscle cells, tropomyosin is implicated to take part in the stabilization of cytoskeleton by actin binding and regulating the microfilament arrangement. In case of chicken, it can be found in the mucous membrane of the small intestine (Xie, Miyazaki & Hirabayashi, 1991). Tropomyosin isoforms in the gastrointestinal tract are known to be responsible for the polarization of gastrointestinal epithelial cells and for stress fiber formation (Dalby-Payne, O’Loughlin & Gunning, 2003). A 2.36-fold increase in tropomyosin alpha-1 (TPM1) level was observed in fasted groups compared to ad libitum group.

### Gene expression analysis

**Figure 3:**
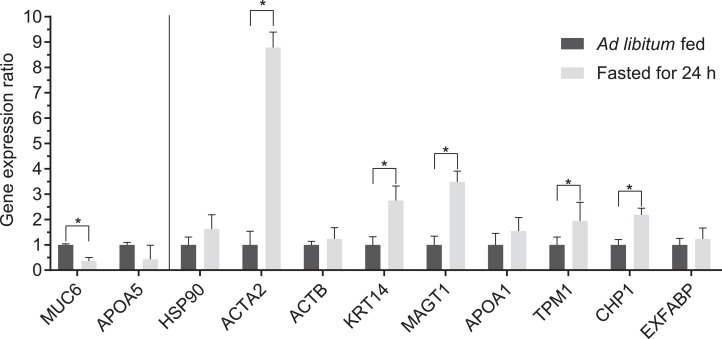
Relative quantification of mRNA of genes encoding the differentially expressed proteins. Relative expression was determined by qPCR and data are presented as group means, error bars represents ± SEM (with *n* = 8 in each group). *Indicates statistically significant difference between groups (*P* < 0.05). The vertical line separates between downregulated (left) and upregulated (right) protein spots compared to ad libitum fed group.

All the differentially expressed proteins, identified by MS, were analyzed by quantitative real-time PCR for mRNA expression. Results are shown in Fig. 3. Among the investigated transcripts, *MUC6* was showed lower expression, while *ACTA2*, Keratin, type I cytoskeletal 14 (*KRT14*), *MAGT1*, *TPM1* and *CHP1* showed higher expression (*P* < 0.05) in the fasted for 24 h group compared to the ad libitum fed group. These results are in accordance with the observed protein level changes (looking at the direction of changes). In case of other genes, no significant difference was detected in their mRNA expression.

## Discussion

In broiler chicken raising practice, it is commonly that hatched chicks have a delayed access to feed and water for up to 72 h. This can be due to the differences in hatching time in combination with other treatments and transport until to the broiler farm (Willemsen et al., 2010). It has already been extensively studied that early feed deprivation has detrimental effects on the development and later performance of the chicks at the market age (Gonzales et al., 2003; Careghi et al., 2005; Bhanja et al., 2009). These are surely due to the effects of prolonged fasting. Fasting for 18 h or more can significantly decrease the villus height (which through the nutrient absorption occurs) in the duodenum, jejunum and ileum of broiler chickens as well as can cause a decrease in the whole small intestine’s relative weight and length (Gonzales et al., 2003). The chicken small intestine is the main site for nutrient uptake despite little being known about its proteomic alteration during fasting. In this work, we identified several proteins with significantly altered expression in the chicken jejunum caused by 24 h long fasting.

Dunel-Erb et al. (2001) found that cytoskeleton of microvilli consisting of microfilaments was disorganized and the microvilli lost their rigidity by fasting. Increased relative protein level of cytoskeletal β-actin and desmin can be found in pig jejunum caused by intrauterine growth restriction (Wang et al., 2008). Beta-actin protein level was increased in our experiment, however, not at mRNA level which might be at connection to promote villus remodeling or might be a result of an increased protein anabolism caused by fasting. Kamata et al. (2018) also found increased protein levels of several structural proteins (e.g., actin, annexin, collagen, keratin, moesin) in the peripheral organs of mice fasted for 24 h. Keratins are important proteins for stress protection in the colonic epithelium (Asghar et al., 2015) and their expression is upregulated after different stress sources (Helenius et al., 2016). Increased expression of *KRT14* was observed in our experiment and it may be in connection with the increased stress caused by fasting and for the stress protection of villus structure.

Besides changing morphologically under fasting, motility changes of chicken small intestine were also observed previously. Reversed motility can occur to recycle nutritive material between the gastrointestinal segments (Clench & Mathias, 1992). Motility changes of the gastrointestinal tract are driven by smooth muscle contractions. Both smooth muscle and cytoplasmic type actin and tropomyosin alpha-1 chain showed increased expression in the fasted group in our experiment. Proteins encoded by the ACTA2 and TPM1 genes are belonging to the complex required for smooth muscle contraction. In there, alpha actins are the major constituent of the contractile apparatus and tropomyosin 1 is required to regulate the calcium-dependent interaction of actin and myosin during muscle contraction. We hypothesize that the increased expression of these proteins are in connection with the previously found motility changes caused by fasting.

Avian apolipoproteins are expressed in numerous tissues including intestine (Byrnes et al., 1987; Dichlberger et al., 2007). The APOA1 is the main protein constituent of the HDL. Its function is to transport cholesterol from tissues to the liver (reverse lipid transport) across the blood. A significant source of blood plasma APOA1 is the intestines (Glickman & Green, 1977). Overexpression of APOA1 gene can increase the reverse lipid transport in itself (Zhang et al., 2003). After 1 day long fasting, cholesterol content in the chicken HDL’s increases (Peebles et al., 2004). Feed restriction can cause a decrease in APOA1 expression in chicken liver as investigated by Richards et al. (2003). In contrast, expression of APOA1 was found to be increased after 48 h fasting in various mouse tissues (Kamata et al., 2018). In our experiment, an increased expression of APOA1 might be related to the increased homeostatic need for reverse cholesterol transport triggered by 24 h fasting. Gene expression of *APOA5* rises by increasing glucose concentration and also with increasing fatty acid concentration (Guardiola et al., 2012). In contrast with mammals, in case of chicken not only liver but peripheral organs also take part in lipoprotein synthesis. The role of APOA5 in small intestine is lipid uptake, lipoprotein assembly and secretion as suggested by Dichlberger et al. (2007). APOA5 is also a potent plasma triglyceride reducer. Fasting can cause a two-fold decrease in plasma triglyceride level in chickens (Peebles et al., 2004). A decrease in APOA5 protein level was found in case of fasted group which may serve to prevent the further reduction of plasma triglyceride content.

The MUC6 protein is a gel forming mucin required to create the mucosal surface of the gastrointestinal tract. This layer protects against the digestive enzymes and pathogens and also helps to forward the intestinal content. Chicken fasted for 72 h showed a significant villus area decrease together with decrease in mucin layer thickness (Smirnov, Sklan & Uni, 2004), which may affect intestinal function and defense. In our experiment the gel forming MUC6 protein expression decreased in the 24 h fasted group compared to ad libitum state, which suggest a connection with the mentioned phenomenon and a possible decreased intestinal defense function. The HSP90 is conserved from bacteria to vertebrates and a key regulator of protein homeostasis under both physiological and stress conditions (Schopf, Biebl & Buchner, 2017). The chicken jejunal and ileal HSP70 and HSP90 levels can increase in response to heat stress (Varasteh et al., 2015). Increased expression of HPS27 and 90 was also observable along the GIT in pigs fasted for 36 h (Lallès & David, 2011) or days after weaning (Grongnet & David, 2003). Increased expression of HSP90 in the chicken jejunum can indicate an increased stress at the cellular level caused by 24 fasting in our experiment.

### Gene expression analysis

All the differentially expressed proteins identified by MS, were also quantified by qPCR at mRNA level. Among them six also showed statistically significant expression, compared to 2D-DIGE. *ACTA2* was highly transcribed in the fasted group with similar ratio both at mRNA and protein levels (9.97- and 8.79-fold). The gene *MUC6* showed significant decrease in mRNA expression. In case of *APOA5*, *HSP90AA*, *ACTB*, *APOA1*, *EXFABP* no significant difference was detected in mRNA expression. However, not all gene showed significant difference compared to protein levels, but the direction of expressional changes are the same as in case of protein level changes.

### Morphometric analysis

Morphometric analysis did not found any significant changes in case of jejunum. This may be due to low sample number in our case. However, previous investigation using higher biological replicates clearly showed decrease in villus height and area or crypt number in multiple cases (Yamauchi, Kamisoyama & Isshiki, 1996; Geyra, Uni & Sklan, 2001; Gonzales et al., 2003) after fasting.

## Conclusions

All together we found that the 24 h long fasting affects mainly the level of structural and secreted proteins in the jejunum of broiler chicken. According to their biological functions these proteins are cytoskeletal components, involved in fatty acid binding and transport, general stress response and ion- or vesicle transport. All the identified cytoskeletal proteins (ACTA2, ACTB, KRT14, TPM1) showed increased expression suggesting that these proteins might be the most important ones in villus remodeling and the motility alterations caused by fasting. However, some significant changes were observable in case of small intestine morphometric parameters, the low number of samples measured does not allow us to make any strong conclusions linking morphometric changes to the observed proteomics alterations. The other identified proteins (EXFABP, MAGT1, APOA1, APOA5, MUC6, HSP90A) might show increasing protein amounts in the fasted group as a response to the stress produced by the fasting and most probably have protective roles maintaining the homeostasis during fasting.

## Supplemental Information

10.7717/peerj.6588/supp-1 Supplemental Information 1 Sample protein concentrations measured with RC DC Protein assay.

10.7717/peerj.6588/supp-2 Supplemental Information 2 Reference gene with stable expression (TBP) was revealed from five candidates.

10.7717/peerj.6588/supp-3 Supplemental Information 3 Intestinal changes of morphometric parameters in response to different feeding states. ^a–b^Group means with the same letter are not significantly different (*P* > 0.05). Data represent mean ± SEM (n = 3 biological replicate in each group, a single data point was obtained by averaging the 25 technical measurements from each biological replicate).

10.7717/peerj.6588/supp-4 Supplemental Information 4 The recorded gel images.

10.7717/peerj.6588/supp-5 Supplemental Information 5 Raw and normalized spot density data.

## Additional Information and Declarations

### Data Availability

The following information was supplied regarding data availability: Raw data is available in the Supplemental Materials.
