# Supplementary material for: Proteomics alterations in chicken jejunum caused by 24 h fasting
Source: PeerJ. 2019 Mar 26;7:e6588. doi: 10.7717/peerj.6588 (PMC6440466; doi:10.7717/peerj.6588)
Supplement: Supplemental Information 1 [file peerj-07-6588-s001.docx]

|  | **group** | |
| --- | --- | --- |
| **sample** | ***ad lib***^†^ **(µg/µl)** | **F24h**^‡^ **(µg/µl)** |
| 1 | 7.42 | 8.65 |
| 2 | 9.73 | 6.65 |
| 3 | 5.32 | 8.89 |
| 4 | 7.55 | 7.28 |
| 5 | 6.36 | 11.11 |
| 6 | 7.38 | 9.75 |
| 7 | 6.71 | 9.09 |
| 8 | 8.07 | 6.15 |

^†^The *ad libitum* fed group.

^‡^The fasted for 24 h group.
