# Supplementary material for: Proteomics alterations in chicken jejunum caused by 24 h fasting
Source: PeerJ. 2019 Mar 26;7:e6588. doi: 10.7717/peerj.6588 (PMC6440466; doi:10.7717/peerj.6588)
Supplement: Supplemental Information 2 [file peerj-07-6588-s002.docx]

| NoGenes^†^ | *LBR* | *POLR2* | *RPS17* | *TBP* | *YWHAZ* | p95GMV^‡^ |
| --- | --- | --- | --- | --- | --- | --- |
| 1 | 0 | 0 | 0 | 1 | 0 | 0.000343 |
| 1 | 0 | 0 | 0 | 0 | 1 | 0.000397 |
| 1 | 0 | 0 | 1 | 0 | 0 | 0.000548 |
| 1 | 0 | 1 | 0 | 0 | 0 | 0.000625 |
| 1 | 1 | 0 | 0 | 0 | 0 | 0.000916 |

^†^1 indicates that the corresponding gene is included in the subset and 0 that it is not included.

^‡^reference genes ranked by the 95% upper confidence limit for the variance as recommended by Chervoneva et al 2010.
