# Supplementary material for: Proteomics alterations in chicken jejunum caused by 24 h fasting
Source: PeerJ. 2019 Mar 26;7:e6588. doi: 10.7717/peerj.6588 (PMC6440466; doi:10.7717/peerj.6588)
Supplement: Supplemental Information 3 — a–bGroup means with the same letter are not significantly different (P > 0.05). Data represent mean ± SEM (n = 3 biological replicate in each group, a single data point was obtained by averaging the 25 technical measurements from each biological replicate). [file peerj-07-6588-s003.docx]

| **tissue** | **group** | | |
| --- | --- | --- | --- |
| ***duodenum*** | ***ad libitum*** | **F24h** | **F24hRF2h** |
| crypt depth (mm) | 0.15±0.010^a^ | 0.16±0.003^a^ | 0.18±0.005^a^ |
| *m. externa* (mm) | 0.21±0.023^a^ | 0.19±0.002^a^ | 0.21±0.005^a^ |
| villus area (mm^2^) | 0.13±0.013^a^ | 0.11±0.027^a^ | 0.16±0.019^a^ |
| villus length (mm) | 0.74±0.053^a^ | 0.73±0.058^a^ | 0.74±0.063^a^ |
| ***jejunum*** |  |  |  |
| crypt depth (mm) | 0.13±0.003^a^ | 0.12±0.006^a^ | 0.13±0.008^a^ |
| *m. externa* (mm) | 0.14±0.001^a^ | 0.15±0.005^a^ | 0.13±0.008^a^ |
| villus area (mm^2^) | 0.14±0.008^a^ | 0.11±0.003^a^ | 0.12±0.015^a^ |
| villus length (mm) | 1.00±0.091^a^ | 0.74±0.075^a^ | 0.87±0.091^a^ |
| ***ileum*** |  |  |  |
| crypt depth (mm) | 0.09±0.002^a^ | 0.09±0.004^a^ | 0.10±0.004^a^ |
| *m. externa* (mm) | 0.15±0.003^a^ | 0.15±0.002^a^ | 0.16±0.012^a^ |
| villus area (mm^2^) | 0.06±0.002^a^ | 0.06±0.004^ab^ | 0.09±0.002^b^ |
| villus length (mm) | 0.66±0.109^a^ | 0.58±0.054^a^ | 0.63±0.023^a^ |

^a-b^Group means with the same letter are not significantly different (*P* > 0.05). Data represent mean ± SEM (n = 3 biological replicate in each group, a single data point was obtained by averaging the 25 technical measurements from each biological replicate).
