# Supplementary figures and images for: Proteomics alterations in chicken jejunum caused by 24 h fasting
Source: PeerJ. 2019 Mar 26;7:e6588. doi: 10.7717/peerj.6588 (PMC6440466; doi:10.7717/peerj.6588)

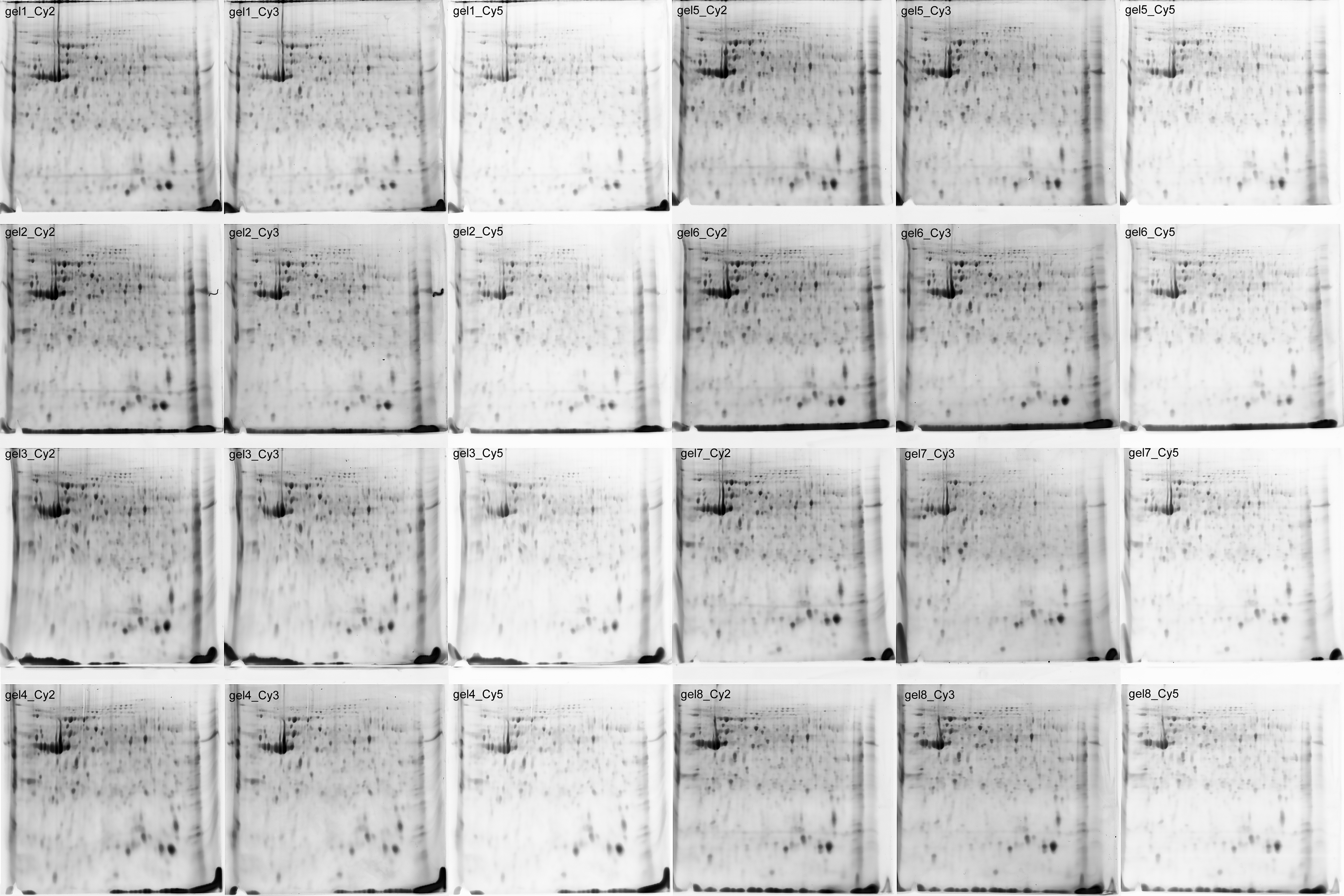

Supplement: Supplemental Information 4 [file peerj-07-6588-s004.png]
